# Molecular basis of primary immune deficiencies in a highly inbred population

**Published:** 2011-01-10

**Authors:** MR Barbouche, I Ben Mustapha, F Mellouli, N Dhouib, R Riahi, K Ben Farhat, H Ouadhani, M Bejaoui, K Dellagi

**Affiliations:** 1 Department of Immunology, Institut Pasteur de Tunis, Tunis, Tunisia; 2 Bone Marrow Transplantation Center, Tunis, Tunisia

## 

Primary immune deficiencies are a heterogeneous group of inherited disorders in which dysfunctions of the immune system cause an enhanced susceptibility to infections. Their frequency is higher in Tunisia and the Maghreb as compared to European and North American countries. Indeed, our population is characterized by a high frequency of consanguineous marriages and this may account for the higher incidence of autosomal recessive primary immune deficiencies observed.

During the last fifteen years, we have been able as a reference center in Tunisia to establish the diagnosis of primary immunodeficiency in 397 patients including 12 patients from Algeria and Libya. The immunological investigation including phenotypic analysis of peripheral blood cells, lymphocyte proliferation assays, phagocytic cells functions and measurement of immunoglobulins as well as complement allowed us to classify these cases in combined immune deficiencies (27%), predominantly antibody deficiencies (23%), phagocytic cells defects (23%), complement deficiencies (1%), other well-defined syndromes (24%) and other immunodeficiencies (2%). The study of the molecular basis of these diseases in our settings as compared to other European, North American and Asian studies shows a high incidence of autosomal recessive diseases including Ataxia-telangiectasia, Bare Lymphocyte syndrome, Mendelian susceptibility to mycobacteria and Leukocyte adhesion deficiency. Moreover, we have observed a higher frequency of autosomal recessive forms of classically X-linked immune deficiencies such as autosomal recessive agammaglobulinemia and chronic granulomatous disease. A founder effect in our population with a single gene mutation accounting for most cases has been observed for several of these primary immune deficiencies.

The establishment of an accurate diagnosis allowed the appropriate treatment of these patients according to the cellular and molecular basis of the disease by bone marrow transplantation, substitutive immunoglobulin therapy, IFNg therapy...etc. The genetic study was aimed: first, to allow an appropriate genetic counselling and a prenatal diagnosis in these consanguineous affected families; second, to help unravelling the immune system functioning since these particularly rare autosomal forms of immune deficiencies offer a unique physiopathological model to study specific host defense pathways in humans.

